# Functional Relevance of CTLA4 Variants: an Upgraded Approach to Assess CTLA4-Dependent Transendocytosis by Flow Cytometry

**DOI:** 10.1007/s10875-023-01582-9

**Published:** 2023-09-23

**Authors:** Jessica Rojas-Restrepo, Elena Sindram, Simon Zenke, Hanna Haberstroh, Noriko Mitsuiki, Annemarie Gabrysch, Katrin Huebscher, Sara Posadas-Cantera, Máté Krausz, Robin Kobbe, Jan C. Rohr, Bodo Grimbacher, Laura Gámez-Díaz

**Affiliations:** 1 Institute for Immunodeficiency, Medical Center, Faculty of Medicine, University of Freiburg, Freiburg, Germany; 2 Center for Chronic Immunodeficiency, Medical Center, Faculty of Medicine, University of Freiburg, Freiburg, Germany; 3 Faculty of Biology, University of Freiburg, Freiburg, Germany; 4 Spemann Graduate School of Biology and Medicine (SGBM), University of Freiburg, Freiburg, Germany; 5 Present Address: Matterhorn Biosciences GmbH, Basel, Switzerland; 6 Department of Rheumatology and Clinical Immunology, University Medical Center Freiburg, Freiburg, Germany; 7 Institute for Infection Research and Vaccine Development (IIRVD), University Medical Center Hamburg-Eppendorf, Hamburg, Germany; 8 Present Address: Novartis Institutes for Biomedical Research (NIBR), Novartis Pharma AG, Basel, Switzerland; 9 German Center for Infection Research (DZIF), Satellite Center Freiburg, Freiburg, Germany; 10 CIBSS – Center for Integrative Biological Signaling Studies, University of Freiburg, Freiburg, Germany; 11 RESIST – Cluster of Excellence 2155 to Hanover Medical School, Satellite Center Freiburg, Freiburg, Germany; 12 Department of Infectious Disease Epidemiology, Bernhard Nocht Institute for Tropical Medicine, Hamburg, Germany

**Keywords:** CTLA4, LRBA, transendocytosis, diagnostics, inborn errors of immunity

## Abstract

Variants of uncertain significance (VUS) in *CTLA4* are frequently identified in patients with antibody deficiency or immune dysregulation syndromes including, but not limited to, patients with multi-organ autoimmunity and autoinflammation. However, to ascertain the diagnosis of CTLA4 insufficiency, the functional relevance of each variant needs to be determined. Currently, various assays have been proposed to assess the functionality of *CTLA4* VUS, including the analysis of transendocytosis, the biological function of CTLA4 to capture CD80 molecules from antigen presenting cells. Challenges of this assay include weak fluorescence intensity of the internalized ligand, poor reproducibility, and poor performance upon analyzing thawed cells. In addition, the distinction of pathogenic from non-pathogenic variants and from wild-type *CTLA4*, and the classification of the different VUS according to its level of CTLA4 dysfunction, would be desirable. We developed a novel CD80-expressing cell line for the evaluation of CD80-transendocytosis and compared it to the published transendocytosis assay. Our approach showed lower inter-assay variability and better robustness regardless the type of starting material (fresh or thawed peripheral mononuclear cells). In addition, receiver operating characteristic analysis showed 100% specificity, avoiding false positive results and allowing for a clear distinction between pathogenic and non-pathogenic variants in *CTLA4*-variant carriers. With our transendocytosis assay, we assessed the pathogenicity of 24 distinct *CTLA4* variants from patients submitted to our diagnostic unit. Significantly impaired transendocytosis was demonstrated for 17 *CTLA4* variants, whereas seven variants tested normal. In conclusion, our upgraded transendocytosis assay allows a reliable assessment of newly identified variants in *CTLA4*.

**Supplementary Information:**

The online version contains supplementary material available at 10.1007/s10875-023-01582-9.

## Introduction

Cytotoxic T-lymphocyte-antigen 4 (CTLA4) is an essential negative immune regulator constitutively expressed on regulatory T cells and upregulated on activated T cells [1]. Upon T cell receptor (TCR) activation, intracellular CTLA4-containing vesicles are rapidly mobilized to the surface of T cells to form homodimers that outcompete CD28 for its binding to the co-stimulatory molecules CD80 and CD86 (also known as B7-1 and B7-2, respectively), which are expressed on antigen-presenting cells (APC). Following its binding, CTLA4 removes B7 molecules from the surface of APCs and CTLA4:B7 complexes are internalized into the regulatory T cells by a mechanism known as transendocytosis [2]. Thus, CTLA4 controls the availability of B7 molecules, playing a pivotal role in maintaining peripheral tolerance and controlling T cell–driven immune response [1].

Germline mutations in human *CTLA4* lead to an autosomal dominant syndrome with incomplete penetrance [3, 4] known as CTLA4 insufficiency. This syndrome is characterized by lymphoproliferation, autoimmune lymphocytic infiltration of non-lymphoid organs, autoimmune cytopenias, a poor humoral response, and an elevated risk of malignancies [3–6]. Currently, the diagnosis of CTLA4 insufficiency relies on the clinical presentation and the detection of genetic variants in *CTLA4*. However, the biological impact of a monogenic defect cannot be ascertained solely by genetic sequencing. Any novel genetic change requires further experimental evidence to clarify whether the identified genetic variant has functional and biological consequences that may underlie the clinical phenotype.

Several methods have been described to assess *CTLA4* variants of uncertain significance (VUS), including the analysis of CTLA4 protein expression (at the cell surface and/or intracellular), Treg suppression, and CD80/CD86 transendocytosis [7, 8]. However, these tests can be challenging, since CTLA4 protein expression is not always affected in patients with point mutations in *CTLA4* [8–10], and Treg functionality assays can be impacted by the low Treg numbers frequently observed in *CTLA4*-variant carriers [11]. The transendocytosis assay was developed in 2009 and comprises the evaluation of CD80/CD86 internalization in patients’ regulatory T cells after co-culturing them with Chinese hamster ovary (CHO) cells, engineered to express human CD80/CD86, thereby functioning as artificial APC. It unraveled the extrinsic function of CTLA4 [2, 7] and is a frequently used method as it directly examines the functional impact of *CTLA4* variants [4, 5, 9, 12]. The test is also suitable to evaluate the biological impact of biallelic mutations in the lipopolysaccharide-responsive beige-like-anchor-protein (*LRBA*), since LRBA facilitates the recycling of CTLA4 to the cell surface [13]. Therefore, the transendocytosis assay has been implemented in our diagnostic unit at the Center for Chronic Immunodeficiency (CCI) in Freiburg. In our hands, the initially available CD80-GFP expressing cell line showed weak intensities of the fluorescent protein once internalized into the regulatory T cells, impeding the distinction of pathogenic from non-pathogenic *CTLA4* variants or from wild-type *CTLA4*. In addition, we frequently experienced variable or non-reproducible results when thawed cells were used as starting material. We therefore sought for optimizations of the diagnostic approach, aiming at facilitating the classification of novel VUS in *CTLA4* and quantification of the associated severity, thereby possibly establishing a rationale for the huge clinical variability seen in this monogenetic condition.

In this study, we replaced the established CD80-GFP expressing CHO cells with a novel CD80-mScarlet expressing CHO cell line and compared the accuracy and the inter-assay variability of the transendocytosis assay using isolated CD4+ T cells from patients with heterozygous mutations in *CTLA4* or biallelic mutations in *LRBA*. In addition, we calculated the receiver operating characteristic (ROC) of the transendocytosis assay and performed a correlation analysis using the CTLA4 haploinsufficiency (CHAI) morbidity score [14] as a clinical score of disease severity. We provide an upgraded approach to assess CD80-transendocytosis by flow cytometry which facilitates the assessment of the pathogenic impact of VUS in *CTLA4.*

## Materials and Methods

### Sample Collection

Peripheral blood mononuclear cells (PBMCs) were extracted from blood samples of healthy donors (HD) and patients harboring *CTLA4* mutations by density gradient centrifugation using Lymphoprep^TM^ (Axis-Shield), according to the manufacture’s protocol. PBMCs were either tested immediately for CD80-transendocytosis or frozen in freezing medium (80% fetal calf serum [FCS] and 20% dimethyl sulfoxide [DMSO]) and stored in liquid nitrogen until use. This study was conducted under the following ethics protocols: vote no. 295/13 version 200149 and vote no. 60/18 of the ethics committee of the University of Freiburg, Germany. All patients signed a written consent to participate in our study according to local ethics committee guidelines.

### CTLA4 Expression in Regulatory T cells

Freshly isolated or thawed PBMCs were cultured as 2×10^5^ cells in 96-well round-bottomed plates in RPMI medium (10% FCS, 1 μg/ml penicillin, and 1 μg/ml streptomycin), and incubated for 16 h in the presence or absence of Dynabeads^TM^ T-Activator CD3/CD28 (Invitrogen) at a ratio of 1:1 (beads:cells). After incubation, cells were either stained for surface CTLA4 expression or intracellular CTLA4 expression. For surface CTLA4 expression, cells were stained with anti-CD4-PercPCy5.5 (Invitrogen), anti-CD45RO-PECy7 (Invitrogen), and anti-CTLA-4-BV421 (BNI3, BD Bioscience), while for intracellular CTLA4 expression cells were stained only with anti-CD4-PercPCy5.5 (Invitrogen) and anti-CD45RO-PECy7 (Invitrogen). Following fixation and permeabilization, intracellular staining using either only anti-FOXP3-PE/FITC (PCH101, Invitrogen) (surface CTLA4 expression) or anti-FOXP3-PE/FITC (PCH101, Invitrogen) and anti-CTLA-4-BV421 (BNI3, BD Bioscience) (intracellular CTLA4 expression) was performed. Cells were acquired on a BD LSRFortessa™ cytometer and analyzed using FlowJo^TM^ 7.6.5 Software (TreeStar Inc., USA).

### Transendocytosis Assay

CD80-transendocytosis was assessed by flow cytometry as described previously [4]. Briefly, primary human CD4^+^ T cells from patients and HD were purified from PBMCs by negative selection using CD4^+^ T cell-enrichment cocktail and microbeads kit (Miltenyi^TM^). Isolated CD4^+^ T cells were cultured in 96-well round-bottomed plates and stimulated with Dynabeads^TM^ T-Activator CD3/CD28 (Invitrogen) at a ratio of 1:1 (beads:cells) for 16 h in the presence of CHO cells stably expressing either CD80-GFP [2] or CD80-mScarlet. To inhibit lysosomal degradation, 40nM of Bafilomycin A (Invivogen) was added to the cells. After 16 h of incubation at 37°C, cells were stained for extracellular markers with anti-CD4-PercPCy5.5 (Invitrogen) and anti-CD45RO-PECy7 (Invitrogen). Following fixation and permeabilization, cells were stained with anti-FOXP3-PE/FITC (PCH101, Invitrogen) and anti-CTLA-4-BV421 (BNI3, BD Bioscience) and acquired on a BD LSRFortessa™ cytometer. Cells were gated on CD4^+^CD45RO^+^FOXP3^+^ and analyzed for GFP or mScarlet uptake. The GFP signal was measured in the 488-nm channel using the 530/30 band pass filter, while the mScarlet signal was quantified in the 561-nm channel using a 586/15 band pass filter. All samples (GFP or mScarlet) were always analyzed at the same cytometer under the same settings. Data analysis and calculation of the geometric mean fluorescence intensity (MFI) values were performed using FlowJo^TM^ 7.6.5 Software (TreeStar Inc., USA). The optimal cutoff values for the transendocytosis assay were determined based on the maximum sensitivity and specificity values given by the ROC curve analysis using GraphPad prism software.

### CD80-Expressing CHO Cells

CHO cells stably expressing CD80-mScarlet were generated by retroviral transduction with pMIG-plasmids encoding mScarlet-tagged human CD80 and CD86. Virus-containing supernatants were collected 24 h and 48 h after Fugene6-based (Promega) transfection of retroviral plasmids into Platinum-A packaging cells (Cell Biolabs). CHO cells (purchased from ATCC) were plated on Retronectin (20μg/ml, Takara)-coated non-tissue culture treated 24-well flat-bottom plates (Greiner) in IMDM, 10% FCS (PAN Biotech), l-glutamine (Thermo Fisher Scientific), Pen/Strep (Thermo Fisher Scientific), 50 μM ß-Mercaptoethanol (Thermo Fisher Scientific), and spin-infected with virus-containing supernatants (2000 rpm, 30°C, acc 3, no brake). Transduced CD80-mScarlet CHO cells were sorted by flow cytometry. CHO cells expressing CD80-GFP have previously been published [2] and were kindly provided by Professor David Samson (UCL, London).

### CTLA4 Haploinsufficiency Morbidity Score (CHAI)

The CHAI morbidity score is a disease assessment score that was developed based on the data of the first 130 CTLA4 insufficiency patients analyzed [5] and an additional 73 unpublished patients. The CHAI morbidity score quantifies organ involvement by integrating specific laboratory values including FACS results, imaging data, and physiological functional results into a score and is described in detail in [14].

### Statistical analysis

Data were analyzed using GraphPad Prism software version 8. Results are presented as mean ± standard deviation (SD). The Mann-Whitney *U* test was used to compare differences between two groups with a confidence level of 95%. *p* values <0.05 were considered statistically significant (*p<0.05; **p<0.01; ***p<0.001; ****p<0.0001). The coefficient of variation (CV) values for the transendocytosis assay were calculated as follows: CV= (standard deviation/mean) × 100%.

## Results

### Improved Robustness of CTLA4 Transendocytosis Assay Using CD80-mScarlet CHO Cells

**Fig. 1:**
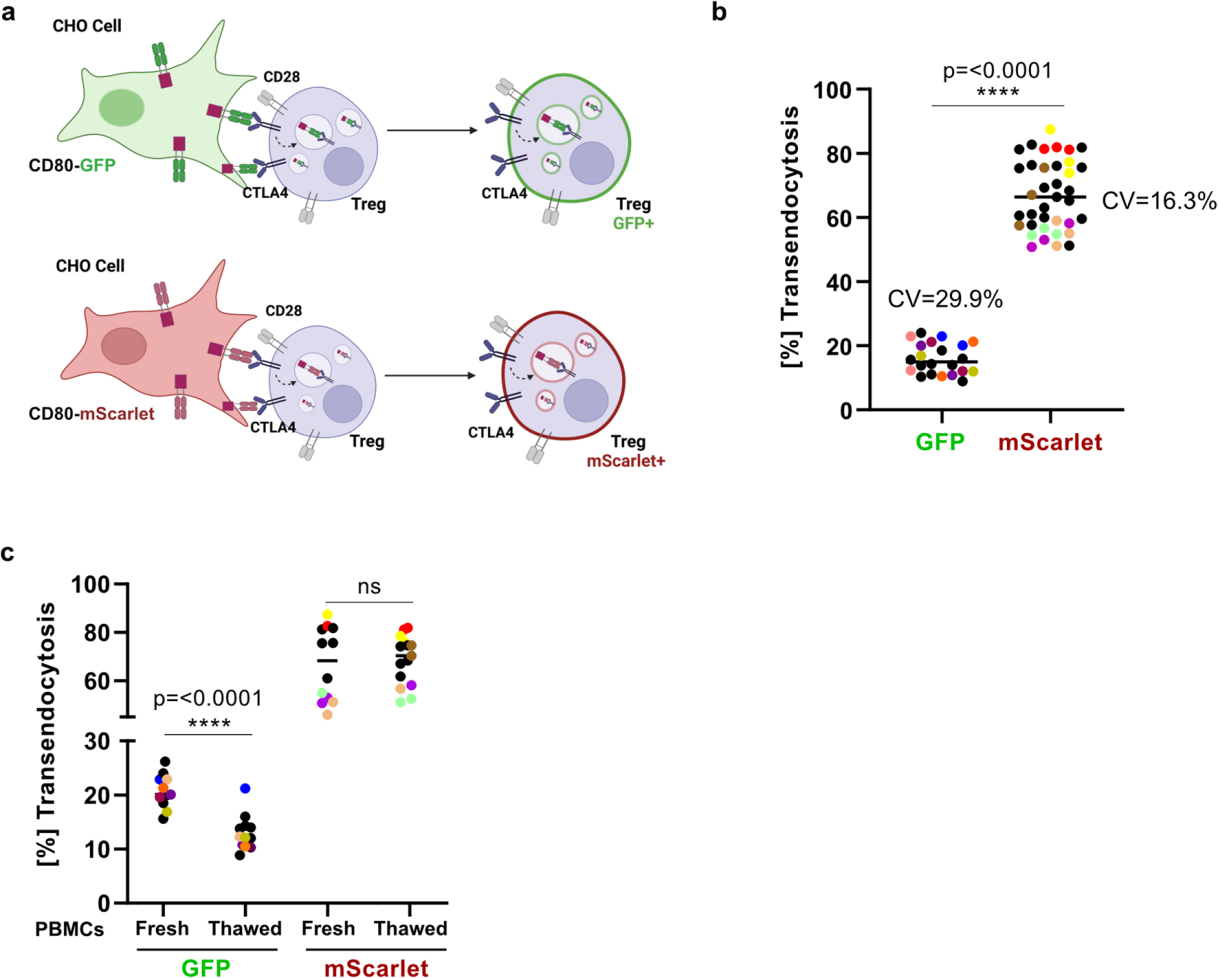
The CTLA4 transendocytosis assay shows higher robustness when using CD80-mScarlet CHO cells. **a** Schematic illustration of the transendocytosis process. Human CD4+ T cells are co-cultured for 16h with CHO cells expressing CD80 tagged with either GFP (top panel) or mScarlet (bottom panel). After CTLA4-CD80 engagement, CTLA4 removes and internalizes the fluorescent tagged CD80- protein. Fluorescent CD80 is subsequently detected in CD4^+^CD45RO^+^FOXP3^+^ T cells by flow cytometry. **b**–**c** Percentage of transendocytosis in activated CD4^+^FOXP3^+^ regulatory T cells from healthy donors (HD) (GFP, *n*=10; mScarlet, *n*=12) showing the inter-assay variability percentage; in **b** total number of samples and **c** fresh and thawed PBMCs. CD80-GFP/fresh PBMCS (*n*=7), CD80-GFP/thawed PMBCS (*n*=9); CD80-mScarlet/fresh PBMCS (*n*=8) and CD80-mScarlet/thawed PMBCS (*n*=10). All data shown in this figure are from ≥3 independent experiments with 2–4 biological replicates. Color-coded circles in CD80-GFP and -mScarlet represent replicates per individual. *p*-values were calculated using the Mann–Whitney test; the horizontal lines in **b** and **c** represents the median values. The coefficient of variation (CV) values for the transendocytosis assay were calculated as follows: CV= (standard deviation/mean) × 100%

Upon endocytosis, newly formed endosomes rapidly acidify their lumen, achieving pH levels around 6.5 in early endosomes to below 5 in lysosomes. Ligands acquired by transendocytosis are exposed to these conditions. While tagging ligands with GFP has been frequently used to investigate transendocytosis, GFP is actually ill-suited for this acidic pH that rapidly quenches its fluorescence. Reasoning that using a more acid-stable fluorochrome might improve the performance of transendocytosis assays, we generated a CHO cell line expressing CD80 tagged with mScarlet (Fig. 1a). In order to account for variations in CD80-ligand availability, we conducted a quantitative analysis of CD80 expression in both GFP and mScarlet CHO cells and detected an equal ligand expression (Supplementary Figure 1). We then compared CTLA4-dependent transendocytosis of CD80 in CHO cells expressing CD80-GFP vs. CD80-mScarlet in activated CD4^+^ T cells from 59 HD. Our results showed that the proportion of detectable transendocytosis ranged from 8.9 to 24% (median, 14.9) using CD80-GFP and from 50.8 to 87.4% (median, 66.4) using CD80-mScarlet (Fig. 1b). The inter-assay coefficient of variation (CV) was 29.9% versus 16.3% for the GFP- vs. mScarlet-based assay, respectively (Fig. 1b). These results indicate that the CD80-mScarlet-based assay was able to detect transendocytosis in more cells and with a lower degree of variability. As we had previously observed that performance of the CTLA4 transendocytosis assay can be reduced when applying it to thawed cells, we set out to test this in HD using the newly generated CD80-mScarlet CHO cells. A median of 12% vs. 20% of thawed versus freshly isolated T cells, respectively, acquired CD80-GFP. In contrast, for CD80-mScarlet, the medians were 70% vs. 68% for thawed versus freshly isolated T cells, respectively. In summary, CD80 acquisition *via* transendocytosis can be evaluated using both types of fluorescent proteins. However, the mScarlet-based assay exhibited (i) less overall inter-assay variability, (ii) strong CD80-fluorescence signal after internalization, and (iii) comparable results independent of the starting material. Since CTLA4 binds to the B7 molecules with distinct affinity and avidity (CTLA4:CD80 0.2μM and CTLA4:CD86 2μM) [15], we also examined whether basing the assay on CD80 or CD86 would have an impact on the proportion of transendocytosed ligand. We therefore developed new CHO cell lines expressing CD80 or CD86 either tagged with GFP or mScarlet. Our results showed no significant difference in the percentage of CTLA4-positive cells from HD (*n*=14) acquiring CD80 or CD86 (Supplementary Figure 2). Interestingly, our newly generated CHO GFP-based cell lines showed an overall increased percentage of transendocytosis; however, the inter-assay %CV was higher when using the GFP-based cell lines (%CV CD80-GFP=31.89; %CV CD86-GFP=34.83) compared to mScarlet-based cell lines (%CV CD80-mScarlet=14.48; %CV CD86-mScarlet=11.66), regardless of the expressed co-stimulatory molecule.

### CTLA4 Transendocytosis Using CD80-mScarlet CHO Cells Facilitates the Identification of Patients with Functional CTLA4 Defects

**Fig. 2:**
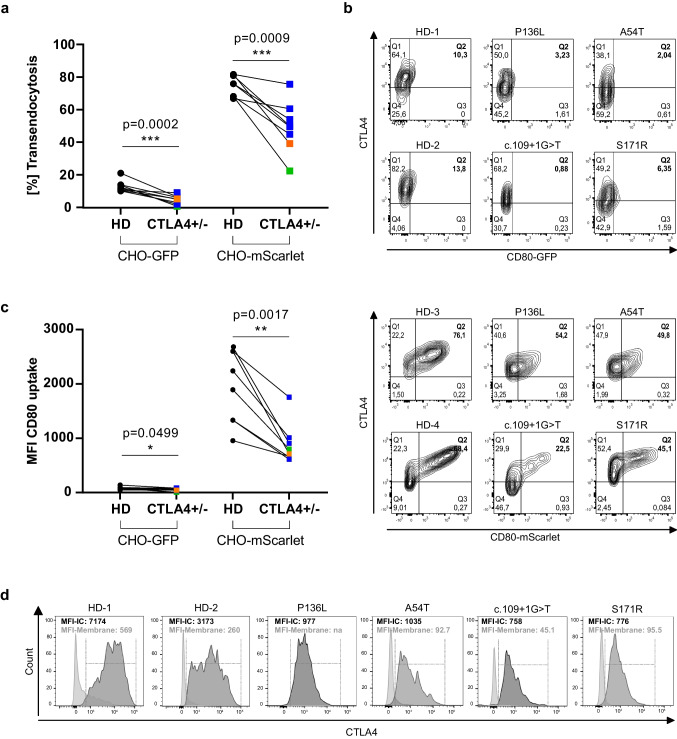
CTLA4 transendocytosis using CD80-mScarlet CHO cells facilitates the identification of patients with functional CTLA4 defects. **a** Percentage of transendocytosis in stimulated CD4^+^FOXP3^+^ regulatory T cells from HD (*n*=8) and *CTLA4*-variant carriers (*n*=8), co-cultured with CHO cells expressing CD80-GFP or CD80-mScarlet. **b** Representative flow cytometry plots depicting the percentage of transendocytosis of CD80-GFP (top panel) or CD80-mScarlet (bottom panel) of HD versus patients carrying unique heterozygous mutations in *CTLA4.* **c** Mean fluorescence intensity (MFI) of internalized CD80-GFP or -mScarlet in regulatory T cells from HD versus patients. Type of mutations are color-coded: blue indicates missense; orange indicates frameshift, and green indicates splice-site variants. **d** Histograms show overlay of surface CTLA4 expression (light gray) and total intracellular CTLA4 (dark gray) in activated CD4^+^FOXP3^+^ regulatory T cells. *p*-values were calculated using the Mann–Whitney test

Next, we analyzed the percentage of transendocytosis in cells derived from eight *CTLA4*-variant carriers and compared them to HD using the two CHO cell lines expressing either CD80-GFP or CD80-mScarlet. Both cell lines revealed reduced proportions of CD4^+^ T cells acquiring CD80 in all patients analyzed compared to HD (Fig. 2a). However, the visibility of the internalized CD80-mScarlet by CTLA4-positive recipient cells was increased compared to CD80-GFP (contour plots Q2, Fig. 2b). In addition, the percentage of cells undergoing transendocytosis was generally higher with the mScarlet-fused CD80, and the difference of the mean fluorescent intensities (MFI) became immediately apparent (HD median 76.1%; median MFI 2068; *CTLA4*-variant carriers median 50.0%; median MFI 756, Fig. 2c). In contrast, smaller differences between HD and *CTLA4*-variant carriers in these parameters, as well as an arduous visual distinction of transendocytosed CD80, were observed when using CD80-GFP (Fig. 1a, b, c). Taken together, we found that CHO cells expressing CD80-mScarlet allow for an improved visualization of the transendocytosis processes and enabled us to determine the degree of impaired transendocytosis associated with distinct *CTLA4* mutations.

Since mutations in *CTLA4* may affect its expression and/or its interaction with ligands, both of which potentially impair transendocytosis, we compared the impact of different *CTLA4* mutations on protein expression in *CTLA4*-variant carriers and HD. As expected, variable intracellular CTLA4 expression levels were observed, which correspond to the percentage of transendocytosis in patient-derived cells. Particularly, patients carrying the P136L, A54T, c.109+1G>T, or S171R mutations, which had reduced intracellular CTLA4 expression and reduced percentage of transendocytosis compared to HD (Fig. 2d). Thus, our findings corroborate that reduced CTLA4 protein levels may be the cause of the impairment in transendocytosis.

### The Diagnostic Performance of CD80-mScarlet and CD80-GFP Expressing CHO Cells in the CTLA4 Transendocytosis Assay Is Comparable

**Fig. 3:**
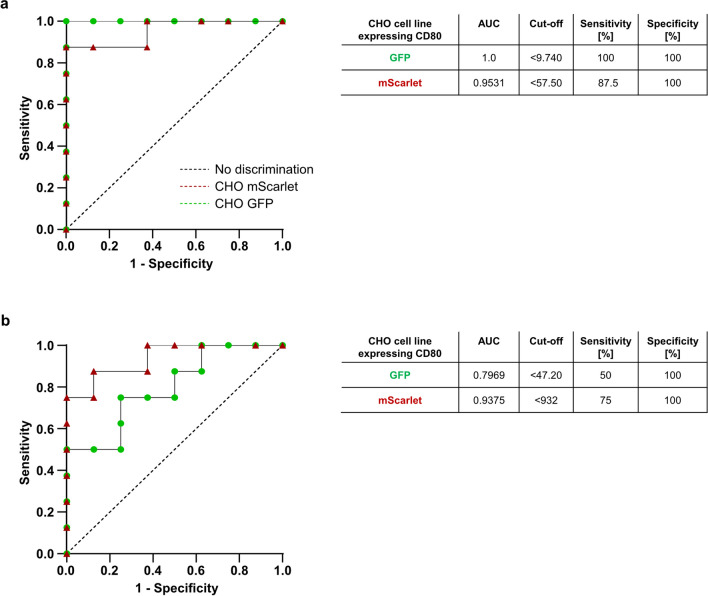
Both CD80-CHO cell lines show comparable receiver operating characteristics (ROC). ROC characteristics curve plot of performance of **a** percent of transendocytosis and **b** MFI for the assessment of *CTLA4* variants with uncertain significance using CD80-GFP and CD80-mScarlet CHO cell lines. Cutoff points, areas under the curve (AUC), percentages of sensitivity, and percentages of specificity are depicted in the tables. Green circles represent CD80-GFP CHO cells and dark red triangles represent CD80-mScarlet CHO cells

To determine the reliability of CD80-mScarlet and CD80-GFP CHO cells in the transendocytosis assay for diagnostic purposes of CTLA4-insufficiency, we performed a ROC analysis based on the percentage and MFI of transendocytosis. ROC analysis based on the percentage of double-positive cells (CTLA4-positive and CD80-fluorescent tagged protein) showed minor differences of the area under the curve (AUC) between the two cell lines (CD80-GFP: AUC 1.0, 95% CI=1.000–1.000; CD80-mScarlet: AUC 0.9531; 95% CI=0.8498–1.000) (Fig. 3a). Accordingly, both cell lines exhibited similar results in their sensitivity and specificity values. While CD80-mScarlet CHO cells had 100% specificity and 87.5% sensitivity based on a cutoff of 57.50, CD80-GFP CHO cells had a specificity and sensitivity of 100% with a cutoff of 9.7 (Fig. 3a).

ROC analysis based on the MFI showed a specificity and sensitivity for CD80-mScarlet expressing cells of 100% and 75%, respectively (AUC of 0.9375, 95% CI=0.8219–1.000), and revealed a 100% specificity for CD80-GFP CHO cells (AUC 0.7969, 95% CI= 0.5757–1.000). However, the latter had a limited sensitivity of only 50% (Fig. 3b). Our results demonstrate that *CTLA4-*variant carriers can be distinguished with a high sensitivity and specificity from HD using either of the two CHO cell lines. However, with the CD80-mScarlet cells, the sensitivity and specificity values remained unchanged in both percentage and MFI of transendocytosis.

### Application of Both Transendocytosis Assays to Individuals with Variants in CTLA4

To validate our assays, regulatory T cells from 26 *CTLA4*-variant carriers and 32 HD were analyzed for CD80-transendcytosis using CHO cells expressing either GFP- or mScarlet-tagged proteins. These patients were similarly distributed by gender, 14 (56%) were females and 11 (44%) males. Detailed information from one patient (P012) was not available. The mean age at their clinical diagnosis was 29 (range from 9 to 52), and 39 (range, from 23 to 71) at their genetic diagnosis (Table 1). At the time of the study, 22 patients were alive, whereas three were deceased. Genetic analysis including next-generation sequencing (NGS) and Sanger sequencing identified 24 distinct mutations in *CTLA4*. Among these, 18 missense, four frameshift, and two splice-site variants were detected. The two splice-site variants were located at the splice-site in intron 1, 16 variants were located in the ligand-binding domain, five in the transmembrane domain, and one variant in the intracellular domain (Table 1). Functional transendocytosis data on 14 of these 24 variants has been published as pathogenic, likely pathogenic, or benign [4, 5, 9, 10, 12]. Thus, ten of the variants described here have previously not been reported (Table 1).

**Table 1:** Demographic data of *CTLA4*-variant carriers

Patient ID	Gender	Nationality	Age at the clinical diagnosis (year)	Age at the genetic diagnosis (year)	*CTLA4* mutation	Intracellular CTLA4 expression	% TEA CHO-GFP	Variant classification [cutoff 9.7%]	% TEA CHO-mScarlet	Variant classification [cutoff 57.5%]	Published variant	^§^CHAI score [%]	CHAI classification	Status
P01	F	German	45	56	c.109+1 G>T	Reduced	0.88	Pathogenic	22.5	Pathogenic	[4]	10.42	Mildly affected	Alive
P02	F	Finnish	uk	uk	^*^c.109+2 T>A	Reduced	–	–	45.9	Pathogenic	uk	NA	NA	Alive
P03	M	uk	uk	uk	^*^G52D	Reduced	–	–	17.2	Pathogenic	uk	NA	NA	Alive
P04	M	uk	uk	71.4	A54T	Reduced	2.04	Pathogenic	49.8	Pathogenic	[9]	NA	NA	Dead
P05	M	uk	uk	uk	R70W	Reduced	–	–	30.6	Pathogenic	[4]	NA	NA	Alive
P06	F	German	52	uk	^*^T72P	Reduced	2.39	Pathogenic	–	–	uk	47.37	Severely affected	Alive
P07	M	German	18	24.2	R75Q	Reduced	7.29	Pathogenic	–	–	[5]	17.65	Mildly affected	Alive
P08	F	Canadian	uk	uk	A86V	Reduced	9.18	Pathogenic	60.8	Non-pathogenic	[5]	NA	NA	Alive
P09	M	uk	uk	uk	^*^Y89H	Normal	–	–	58.5	Non-pathogenic	uk	NA	NA	Alive
P10	F	German	24	25	G109E	Reduced	7.58	Pathogenic	–	–	[5]	33.33	Severely affected	Alive
P11	F	German	uk	uk	G109E	Normal	5.11	Pathogenic	75.8	Non-pathogenic	[12]	18.75	Severely affected	Alive
P12	uk	uk	uk	uk	G109E	Normal	–	–	67.3	Non-pathogenic	uk	NA	NA	uk
P13	M	German	38	40.6	L119R	Reduced	6.72	Pathogenic	50.3	Pathogenic	[12]	52.38	Severely affected	Alive
P14	F	Czech	uk	36	M123Ifs*15	Reduced	4.16	Pathogenic	–	–	[9]	NA	NA	Dead
P15	F	uk	uk	uk	^*^I128M	Normal	–	–	70.7	Non-pathogenic	uk	NA	NA	Alive
P16	M	German	uk	uk	^*^V131A	Reduced	3.28	Pathogenic	–	–	uk	45.83	Severely affected	Alive
P17	M	Belgian	uk	40	P136L	Reduced	3.23	Pathogenic	54.2	Pathogenic	[9]	NA	NA	Alive
P18	F	German	uk	32.3	Y139C	Normal	–	–	55.8	Pathogenic	[10]	11.11	Mildly affected	Alive
P19	F	Italian	uk	uk	N145S	Normal	16.9	Non-pathogenic	–	–	[9]	NA	NA	Alive
P20	M	German	22	22.7	T147Rfs*8	Reduced	–	–	30.7	Pathogenic	[12]	42.11	Severely affected	Dead
P21	F	American	uk	uk	^*^P156L	Reduced	–	–	36.7	Pathogenic	uk	NA	NA	Alive
P22	F	German	uk	uk	^*^L163Sfs*24	Reduced	–	–	37.2	Pathogenic	uk	42.86	Severely affected	Alive
P23	F	German	46	46.2	^*^S171R	Reduced	6.35	Pathogenic	45.1	Pathogenic	uk	15.56	Severely affected	Alive
P24	F	uk	20	uk	^*^S172L	Normal	16.4	Non-pathogenic	–	–	uk	6.67	Unaffected	Alive
P25	M	German	16	40.8	F179Cfs*29	Reduced	5.44	Pathogenic	39.4	Pathogenic	[9]	43.75	Severely affected	Alive
P26	M	German	9	uk	T207A	Normal	–	–	65.5	Non-pathogenic	[9]	10.42	Mildly affected	Alive

*F* female, *M* male, *NA* non-available, *uk* unknown

^§^CHAI score classification= ≥20% (severely affected); ≥10% but <20% (mildly affected); <10% (Unaffected)

*Novel variants

**Fig. 4:**
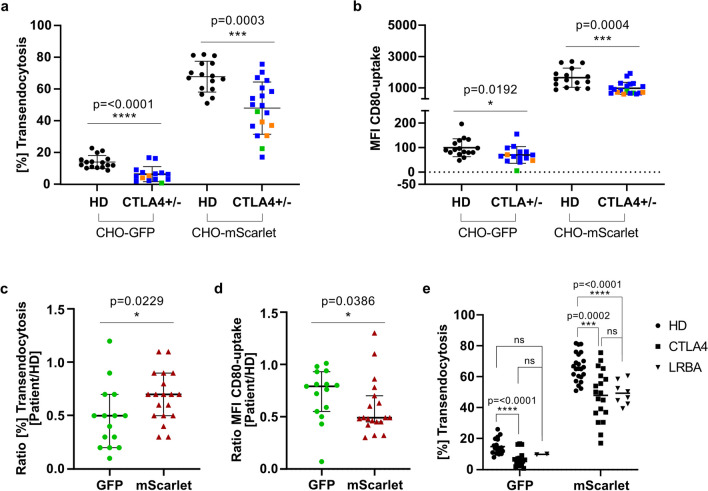
*CTLA4*-variant carriers can be distinguished from healthy donors using both CD80-expressing CHO cells. Dot plots show **a** percentage and **b** MFI of CD80-GFP (*n*=15 *CTLA4*-variant carriers; *n*=16 healthy donors [HD]) and CD80-mScarlet (*n*=19 *CTLA4*-variant carriers; *n*=16 HD) transendocytosis in CD4^+^FOXP3^+^ regulatory T cells from HD and *CTLA4*-variant carriers. Filled circles represent data from HD, filled squares represent data from *CTLA4*-variant carriers. Type of mutations are color-coded: blue indicates missense mutations; orange, frameshift mutations; and green, splice-site mutations. Dot plots show the fold changes of percent of transendocytosis (**c**) and MFI (**d**). Fold change was calculated as a ratio between percent of transendocytosis or MFI values of each *CTLA4*-variant carrier and their matched HD. Green circles represent CD80-GFP CHO cells and dark red triangles represent CD80-mScarlet CHO cells (**e)** Percent of transendocytosis using CD80-GFP (*n*=18) or CD80-mScarlet (*n*=23) CHO cells in regulatory T cells from HD (filled circles), *CTLA4*-variant carriers (CD80-GFP *n*=17; CD80-mScarlet *n*=19; filled squares) and patients with LRBA deficiency (CD80-GFP *n*=2; CD80-mScarlet *n*=8; filled triangles). *p*-values were calculated using the Mann–Whitney test

Consistent with our previous observations (Fig. 2a, c), regulatory T cells from HD and *CTLA4*-variant carriers showed higher frequency and higher MFI of transendocytosis when CD80-mScarlet instead of CD80-GFP CHO cells were used (Fig. 4a, b). An additional ROC curve was calculated for the percentage of transendocytosis of all *CTLA4* variants using CD80-mScarlet CHO cells (Supplementary Figure 3). To account for differences in CTLA4-dependent transendocytosis of CD80, we normalized the percentage of transendocytosis and MFI of CD80-tagged proteins in CTLA4-variant carriers to their matched HD to generate a ratio. Each patient was tested with at least one matched HD in the same assay. Based on a ratio < 0.9 and a cutoff of 57.5% for the percentage of CD80-mScarlet transendocytosis, we classified 13 of the 18 *CTLA4* variants as pathogenic (Fig. 4c, Table 1). Using CD80-GFP CHO cells, we found 12 out of 14 *CTLA4* variants to impair CTLA4 transendocytosis function which was determined by a ratio < 0.9 and a cutoff 9.7% of transendocytosed CD80-GFP (Fig. 4c, Table 1). Six of eight samples that were subjected to both procedures (with mScarlet- and GFP-fused CD80 molecules) were found to be pathogenic by both assays. The variants classified as non-pathogenic (CD80-GFP (*n*=2) and CD80-mScarlet (*n*=5)) showed a ratio ≥ 0.9 of transendocytosis efficiency (Fig. 4c, Table 1)*.* Moreover, a ratio of <0.7 for MFI of transendocytosed CD80 using CD80-mScarlet but not CD80-GFP could differentiate patients with a *CTLA4* variant classified in Table 1 as pathogenic from those with non-pathogenic variants (Fig. 4d). Thus, a ratio < 0.7 of MFI CD80-ligand uptake may be used as an additional parameter to prove the pathogenicity of VUS in *CTLA4*.

LRBA is known to regulate CTLA4 intracellular trafficking by preventing its lysosomal degradation and facilitating its recycling to the plasma membrane of regulatory T cells [13]. Therefore, patients with deleterious biallelic mutations in *LRBA* also show reduced surface CTLA4 expression, explaining the poor control of T cell response *via* CTLA4. This functional link may explain the overlapping clinical phenotype of patients with LRBA deficiency and CTLA4 insufficiency. To examine whether the CTLA4 transendocytosis method is suitable to discriminate patients with CTLA4 insufficiency from LRBA deficiency, we evaluated the percentage of transendocytosis using either CD80-GFP or CD80-mScarlet CHO cells in eight LRBA-deficient patients. We found no difference in percentage of transendocytosis of *CTLA4*-variant carriers (GFP median=5.4%; mScarlet median= 49.8%) compared to LRBA-deficient patients (GFP median=9.9%; mScarlet median, 48.6%). However, with CD80-mScarlet CHO cells, we observed significantly lower percentages of transendocytosis in LRBA-deficient patients compared to HD (median, 48.6% vs. 65.5% in HD) (Fig. 4e), which was not observed with CD80-GFP CHO cells (patients median of 9.9% in patients vs. 13.9% in HD). In conclusion, the CTLA4 transendocytosis method using CD80-mScarlet CHO cells allows the functional verification of LRBA deficiency, but not a distinction between LRBA deficiency and CTLA4 insufficiency.

### The Degree of Impaired CD80 Transendocytosis in CTLA4-Variant Carriers Is Not Dependent on the Affected Amino Acid Position

**Fig. 5:**
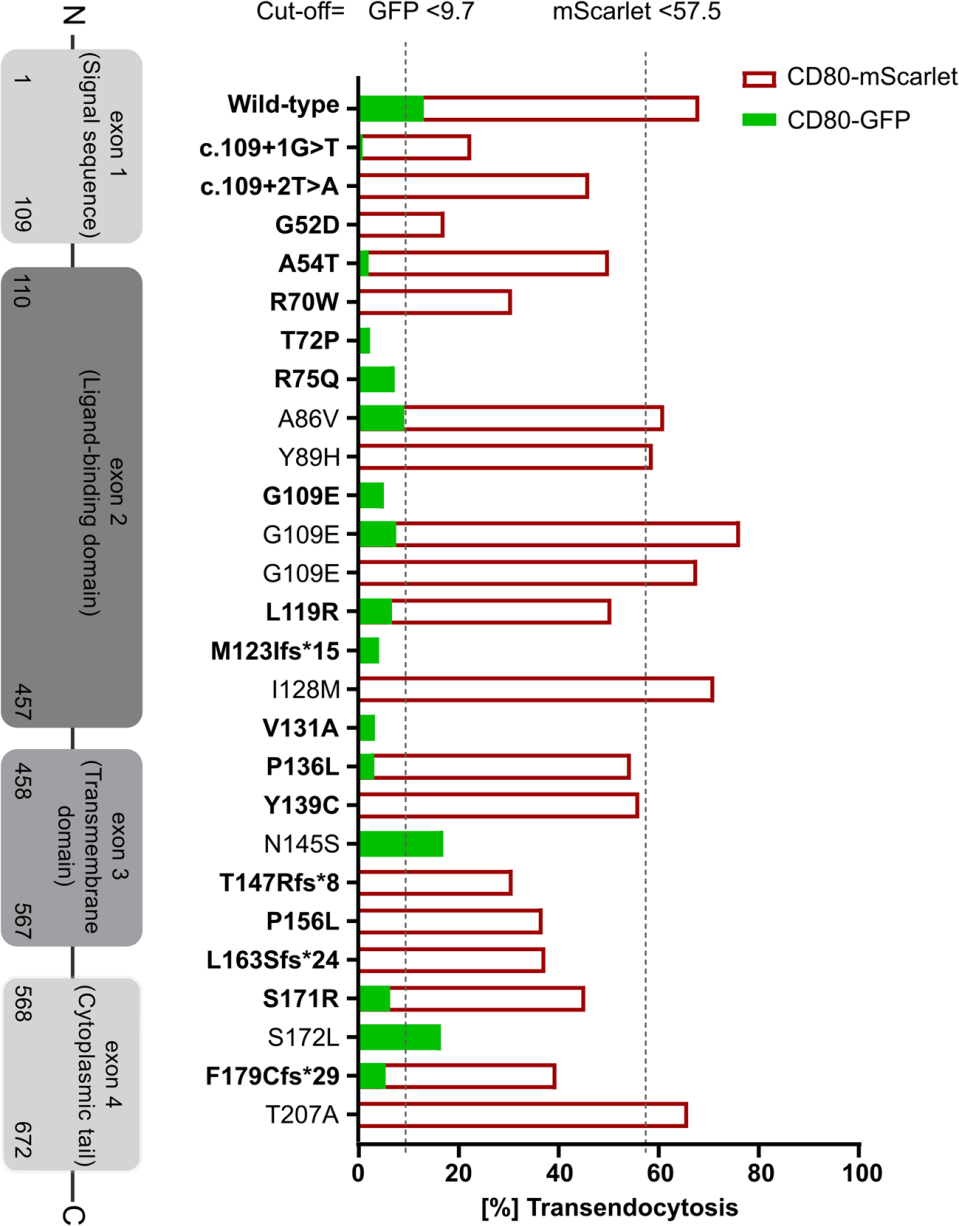
The degree of impaired CD80 transendocytosis in *CTLA4*-variant carriers is not dependent on the affected amino acid position. Functional transendocytosis analysis was performed for 24 *CTLA4* variants, including two splice-site, 18 missense, and four frameshift variants distributed throughout all four exons. Dark red bars represent percent of transendocytosis of CD80-mScarlet and green bars percent of transendocytosis of CD80-GFP. Dotted line indicates the cutoff for the percent of transendocytosis of CD80-GFP and for CD80-mScarlet, respectively

In our cohort study, missense variants in *CTLA4* were the most commonly identified mutations (Table 1). Based on the evaluation of transendocytosis percentages using either CD80-GFP or CD80-mScarlet CHO cells, we observed an impairment of CTLA4 activity in eleven out of 18 missense variants (G52D, A54T, R70W, T72P, R75Q, L119R, V131A, P136L, Y139C, P156L, S171R). Conversely, in seven (A86V; Y89H; G109E; I128M; N145S; S172L; T207A), the transendocytosis percentages were comparable to the HD, suggesting that these variants are non-pathogenic (Fig. 5). Two splice site variants (c.109+1G>T; c.109+2T>A), located in exon one, and four frameshift variants (M123Ifs*15; T147Rfs*8 [located in exon2], L163Sfs*24; F179Cfs*29 [both located in exon 3]) were severely affecting the percentage of transendocytosis (Table 1 and Fig. 5). In addition, we did not find any correlation between the ability to perform transendocytosis and the localization of the respective mutation within the protein sequence (Fig. 5).

**Fig. 6:**
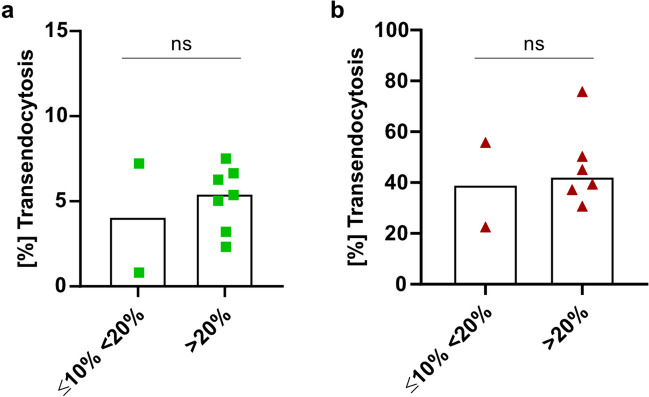
CTLA4-patients with a severe clinical phenotype according to the CHAI score have similarly reduced transendocytosis as mildly affected individuals with the same mutation in *CTLA4.* Bar graphs indicating the percent of transendocytosis in *CTLA4*-variant carriers grouped according to their disease severity. Disease state of *CTLA4*-variant carriers was calculated based on CHAI morbidity score and classified accordingly: ≥20% (severely affected); ≥10%<20% (mildly affected). Green squares represent percent of transendocytosis assay using CD80-GFP CHO cells and dark red triangles represent percent of transendocytosis using CD80-mScarlet CHO cell lines. *p*-values were calculated using the Mann–Whitney test

We then investigated whether *CTLA4*-variant carriers with highly reduced percentage of transendocytosis have a more severe clinical phenotype according to the CHAI morbidity score. Clinical information of 14 patients of our cohort was available. Nine (64.2%) of them were classified as severely affected (CHAI score ≥20%), four (28.5%) as mildly affected (score between 10 and 20%), and one patient was classified as unaffected (CHAI score <10%) (Table 1). We observed no correlation between the percentage of transendocytosis and the CHAI morbidity score, suggesting that the disease severity in CTLA4-insufficient patients is not pre-determined by the efficiency of transendocytosis (Fig. 6).

## Discussion

In recent years, the biology of CTLA4 has been intensively studied. Yet, its precise inhibitory function and its molecular mechanism(s) are incompletely understood. Several/distinct functional models have been proposed including the most-studied transendocytosis of B7 co-stimulatory molecules, where CTLA4 exerts its suppressive function by removing its ligands CD80 and CD86 from the surface of neighboring APCs. Accordingly, the analysis of the transendocytosis ability has become the method of choice, when newly identified genetic variants in *CTLA4*, particularly variants of uncertain significance (VUS), are to be characterized. In this study, we replaced the initially used CD80-GFP CHO cells with CD80-mScarlet CHO cells to assess the degree of impaired transendocytosis in patients with suspected CTLA4 insufficiency or LRBA deficiency.

Using CD80-mScarlet CHO cells, we obtained consistent results regarding the inter-assay CV percentage in both technical and biological replicates. The transendocytosis frequency is sensitive to changes of the cell numbers and cell-to-cell contact [2, 8]. Therefore, technical parameters such as sample quality, CHO cell numbers, and robust fluorescence signals are crucial for a successful approach. In our hands, the transendocytosis ability remained unaffected by the type of starting material (freshly isolated or thawed PBMCs), only when using CD80-mScarlet CHO cells, but was variable with CD80-GFP CHO cells. Since cryopreservation of PBMCs is widely used in clinical and research assays, particularly when patient samples must be shipped over long distances to specialized diagnostic centers, the use of CHO-mScarlet cells is advantageous.

Regarding the overall performance, the percentage of transendocytosis in HD samples varied between 10 and 23% with CD80-GFP, consistent with previous studies [4, 9], but was strongly increased (50.8 to 87.4%) with CD80-mScarlet. One possible explanation is the fluorescence stability of GFP which is subject to environmental conditions. During the transendocytosis process, the CTLA4:CD80 complex is internalized by T cells *via* endocytosis and predominantly localizes in acidic intracellular compartments such as endosomes and lysosomes [16]. Acidic conditions destabilize the GFP chromophore, resulting in a decreased fluorescence quantum yield [17]. In contrast, mScarlet has a higher acid tolerance and is more resistance to acidic environments [17]. Despite the weak signal of the transendocytosed CD80-GFP, patient samples bearing deleterious mutations can be analyzed using GFP. However, a low percentage of transendocytosis may impede the interpretation of the results, particularly when single amino acid changes in CTLA4 are to be analyzed, which often cause a minor reduction of the transendocytosis rate.

Including the MFI values and fold change as part of the data analysis, aiming at overcoming the false positive cases due to low transendocytosis percentages, we found CD80-mScarlet, due to the high fluorescence intensity, advantageous over CD80-GFP.

In addition, we could only observe that distinct *CTLA4* variants inhibit the ability of regulatory T cells to remove CD80 to variable degrees, when CD80-mScarlet CHO cells were used. A recent study reported particular CTLA4 mutations to have a divergent effect on the capacity to accomplish transendocytosis of CD80 or CD86. Cells carrying the CTLA4 variant R70Q preserved their capacity to perform CD80-transendocytosis, whereas a detrimental effect was observed for CD86-transendocytosis [18]. This observation suggests that particular amino acids in CTLA4 selectively interact with CD80, but not with CD86, and *vice versa*. Of note, in regulatory T cells from a patient carrying a different missense mutation at the same amino acid position (R70W), we observed a clear reduction of CD80-transendocytosis. However, we did not pursue further experiments to test whether there is a specific defect in CD86-transendocytosis.

To test the reliability of the newly generated CD80-mScarlet CHO cell line, we analyzed a total of 24 *CTLA4* variants (including ten novel and 14 known variants) in 26 CTLA4+/− patients, and 15 *LRBA* variants in eight LRBA−/− patients. Although both cell lines allow to distinguish between pathogenic and non-pathogenic variants, we observed that patients’ cells carrying the CTLA4 variant G109E could accomplish transendocytosis of CD80 *via* CTLA4 only with CHO-mScarlet cells but not with CHO-GFP cells. The G109E variant in *CTLA4* was first described in 2018 in a severely affected female patient from Germany with a CHAI score of 33.3% [5]. After establishing the transendocytosis assay with CD80-mScarlet CHO cells, revaluation of this patient revealed a comparable percentage of transendocytosis to the HD [12]. Two additional G109E variant carriers also showed normal transendocytosis with CD80-mScarlet CHO cells. Sequence homology alignments showed that glutamic acid (E) occurs at position 109 in various mammalian species, suggesting that G109E does not affect protein function [19]. Consistently, variant effect prediction tools assign G109E to be “possibly benign.” Moreover, as G109E occurs in approximately 0.027% of the healthy population, its disease-causing role remains questionable. However, individuals harboring this variant and presenting with a typical CTLA4 phenotype seem to be over-represented in our IEI cohort—and have also been reported in other cohorts (verbal or written communications). Hence, this variant shall be carefully considered as possibly contributing to the immune phenotype and further immunological analysis of G109E needs to be performed. Further, in order to ascertain the G109E mutation, it would be important to clone the mutation into a vector and analyze its ability to facilitate CD80 and CD86 transendocytosis.

Accurate functional tests are essential to assess rare or novel genetic variants and their disease-causing effect in inborn errors of immunity. Patients with CTLA4 insufficiency often have clinical signs and symptoms that overlap with other IEIs, such as LRBA deficiency [20] or NF-κB1 insufficiency [21, 22], challenging physicians to establish a correct diagnosis. Although the definitive diagnosis of CTLA4 insufficiency and LRBA deficiency relies mainly on genetic studies, additional laboratory tests including CTLA4 expression and transendocytosis should be performed, aiming at facilitating a definitive diagnosis and to determine the pathogenicity of each variant. However, reduction of CTLA4 protein expression is not diagnostic for monogenic *CTLA4*-deficiency on its own, as the binding of the CTLA4 antibody (BNI3) to the CTLA4 protein may be affected by different CTLA4 mutations. This should be considered when interpreting data. Moreover, not only deleterious mutations in *CTLA4* but also mutations in other genes such as *LRBA* may disrupt CTLA4 trafficking and recycling. Nevertheless, the transendocytosis assay has some limitations as a daily practice diagnostic test, including the need of the CHO-cells and trained personal. An alternative to overcome these limitations is to use CD80-Ig to measure CD80-ligand uptake by CTLA4 [8]. This assay developed by Hou et al. uses CD80-Ig instead of CD80-expressing CHO cells, thus evaluating the ability of CTLA4 to bind to CD80 but without assesing the internalization of CD80, which occurs after direct cell-to-cell contact. Importantly, both assays require the establishment of reference data for each laboratory, as sample handling can influence the results. Of note, both assays provide with different information as Hou et al. developed a simplified ligand-binding assay using CD80-Ig instead of CHO cells expressing CD80 coupled to a fluorescent marker (GFP or mScarlet). This assay allows to determine the ability of CTLA4 to bind to CD80, but it does not assess the internalization of CD80, which occurs after direct cell-to-cell contact.

The aim of this work is to provide an improved accurate and robust diagnostic test for clinicians and researchers, to effectively analyze and assess novel variants or VUS in *CTLA4* and *LRBA*. The improved performance of the transendocytosis assay using a CD80-mScarlet CHO cell line allows for discrimination of *CTLA4* mutations based on the percentage of transendocytosis and the MFI with high specificity and sensitivity, and it is therefore recommended to assess *CTLA4* mutations.

### Supplementary Information

Supplementary Fig.1 CD80-ligand expression levels in GFP and mScarlet CHO cells. Blue histograms represent the fluorescence minus 1 (FMO) while the red histograms represent CD80 BV421 staining. (DOCX 320 kb)

Supplementary Fig.2 Comparison of the percent of transendocytosis of newly developed CD80 or CD86 CHO cells tagged to either GFP or mScarlet (developed in our laboratory) in HD (n=14). Open circles represent CHO cells expressing CD80 and filled circles represent CHO cells expressing CD86. *p*-values were calculated using the Mann–Whitney test. The coefficient of variation (CV) values for the transendocytosis assay were calculated as follows: CV= (standard deviation/mean) x 100%. (DOCX 227 kb)

Supplementary Fig.3 Receiver operating characteristics (ROC) analysis of all *CTLA4* variants using CD80-mScarlet CHO cells. (TIF 347 KB)

## Data Availability

The datasets generated during and/or analyzed during the current study are available from the corresponding author on reasonable request.
