# Correction to: Functional Relevance of CTLA4 Variants: an Upgraded Approach to Assess CTLA4‑Dependent Transendocytosis by Flow Cytometry

**DOI:** 10.1007/s10875-023-01596-3

**Published:** 2023-10-09

**Authors:** Jessica Rojas‑Restrepo, Elena Sindram, Simon Zenke, Hanna Haberstroh, Noriko Mitsuiki, Annemarie Gabrysch, Katrin Huebscher, Sara Posadas‑Cantera, Máté Krausz, Robin Kobbe, Jan C. Rohr, Bodo Grimbacher, Laura Gamez‑Diaz

**Affiliations:** 1 Institute for Immunodeficiency, Medical Center, Faculty of Medicine, University of Freiburg, Freiburg, Germany; 2 Center for Chronic Immunodeficiency, Medical Center, Faculty of Medicine, University of Freiburg, Freiburg, Germany; 3 Faculty of Biology, University of Freiburg, Freiburg, Germany; 4 Spemann Graduate School of Biology and Medicine (SGBM), University of Freiburg, Freiburg, Germany; 5 Present Address: Matterhorn Biosciences GmbH, Basel, Switzerland; 6 Department of Rheumatology and Clinical Immunology, University Medical Center Freiburg, Freiburg, Germany; 7 Institute for Infection Research and Vaccine Development (IIRVD), University Medical Center Hamburg-Eppendorf, Hamburg, Germany; 8 Present Address: Novartis Institutes for Biomedical Research (NIBR), Novartis Pharma AG, Basel, Switzerland; 9 German Center for Infection Research (DZIF), Satellite Center Freiburg, Freiburg, Germany; 10 CIBSS – Center for Integrative Biological Signaling Studies, University of Freiburg, Freiburg, Germany; 11 RESIST – Cluster of Excellence 2155 to Hanover Medical School, Satellite Center Freiburg, Freiburg, Germany; 12 Department of Infectious Disease Epidemiology, Bernhard Nocht Institute for Tropical Medicine, Hamburg, Germany

**Correction to: Journal of Clinical Immunology**

10.1007/s10875-023-01582-9

Due to typesetting mistake, there were overlapping values in the first histogram of Figure 2b, which were not there in the proof reading of this figure.

The original version has been corrected.

