# Supplementary material for: Functional Relevance of CTLA4 Variants: an Upgraded Approach to Assess CTLA4-Dependent Transendocytosis by Flow Cytometry
Source: J Clin Immunol. 2023 Sep 23;43(8):2076–89. doi: 10.1007/s10875-023-01582-9 (PMC10661720; doi:10.1007/s10875-023-01582-9)
Supplement: Supplementary file 1 — CD80-ligand expression levels in GFP and mScarlet CHO cells. Blue histograms represent the fluorescence minus 1 (FMO) while the red histograms represent CD80 BV421 staining. (DOCX 320 kb) [file 10875_2023_1582_MOESM1_ESM.docx]

**Supplementary Fig.1** CD80-ligand expression levels in GFP and mScarlet CHO cells. Blue histograms represent the fluorescence minus 1 (FMO) while the red histograms represent CD80 BV421 staining.

**
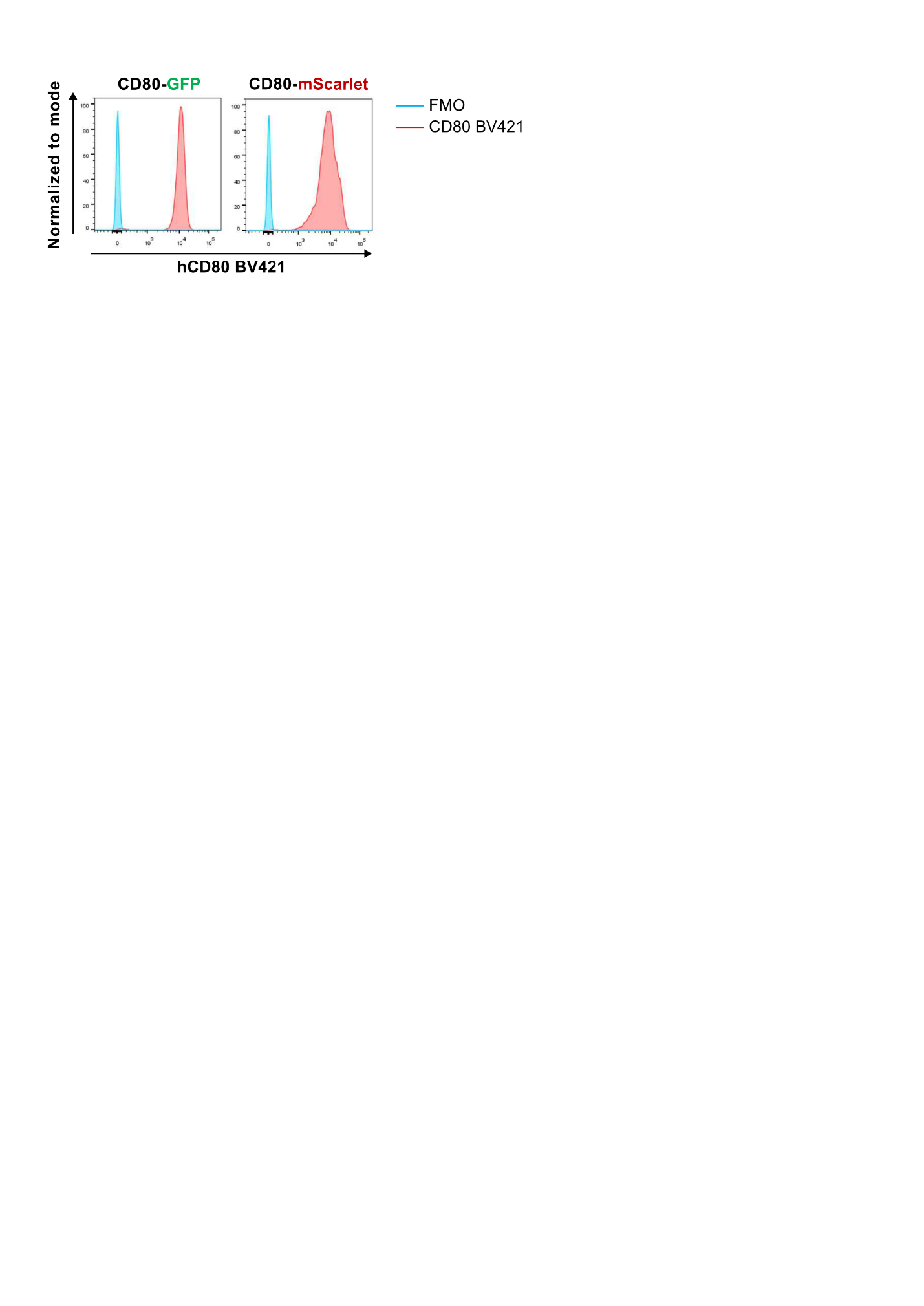
**
