# Supplementary material for: Functional Relevance of CTLA4 Variants: an Upgraded Approach to Assess CTLA4-Dependent Transendocytosis by Flow Cytometry
Source: J Clin Immunol. 2023 Sep 23;43(8):2076–89. doi: 10.1007/s10875-023-01582-9 (PMC10661720; doi:10.1007/s10875-023-01582-9)
Supplement: Supplementary file 2 — Comparison of the percent of transendocytosis of newly developed CD80 or CD86 CHO cells tagged to either GFP or mScarlet (developed in our laboratory) in HD (n=14). Open circles represent CHO cells expressing CD80 and filled circles represent CHO cells expressing CD86. p-values were calculated using the Mann–Whitney test. The coefficient of variation (CV) values for the transendocytosis assay were calculated as follows: CV= (standard deviation/mean) x 100%. (DOCX 227 kb) [file 10875_2023_1582_MOESM2_ESM.docx]

**Supplementary Fig.2** Comparison of the percent of transendocytosis of newly developed CD80 or CD86 CHO cells tagged to either GFP or mScarlet (developed in our laboratory) in HD (n=14). Open circles represent CHO cells expressing CD80 and filled circles represent CHO cells expressing CD86. *p*-values were calculated using the Mann–Whitney test. The coefficient of variation (CV) values for the transendocytosis assay were calculated as follows: CV= (standard deviation/mean) x 100%.

**
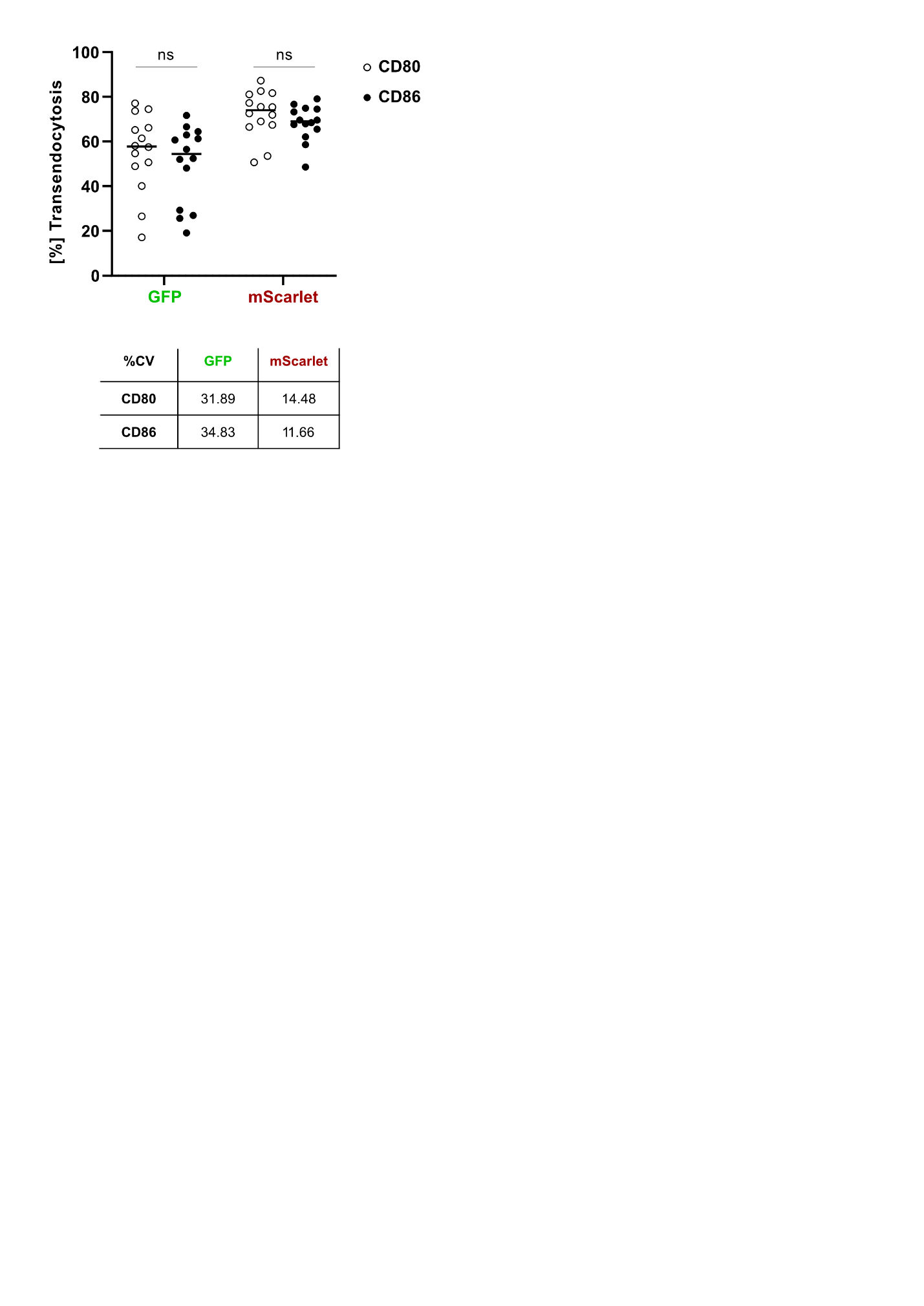
**
