# Supplementary figures and images for: Functional Relevance of CTLA4 Variants: an Upgraded Approach to Assess CTLA4-Dependent Transendocytosis by Flow Cytometry
Source: J Clin Immunol. 2023 Sep 23;43(8):2076–89. doi: 10.1007/s10875-023-01582-9 (PMC10661720; doi:10.1007/s10875-023-01582-9)

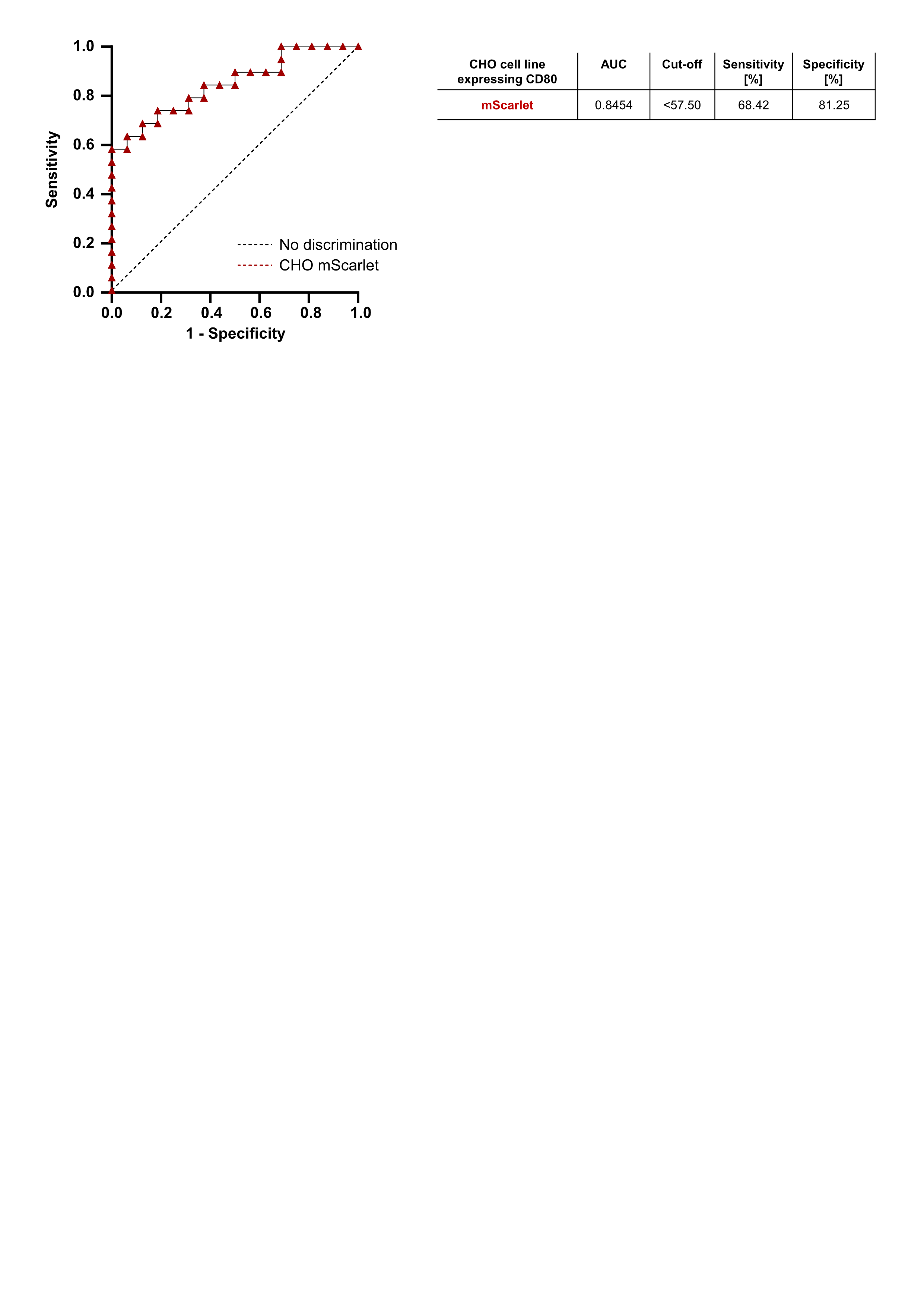

Supplement: Supplementary file 3 — Receiver operating characteristics (ROC) analysis of all CTLA4 variants using CD80-mScarlet CHO cells. (TIF 347 KB) [file 10875_2023_1582_MOESM3_ESM.tiff]
